# Molecular Mechanism of POSTN Mediating M2 Polarization of Kupffer Cells to Promote Hepatic Fibrosis

**DOI:** 10.3390/ph19050752

**Published:** 2026-05-11

**Authors:** Meng-Dan Wang, Shuo-Ying Yuan, Arzu Mijit, Wen Zhang, Yang Wu, Lu-Feng Cheng

**Affiliations:** 1 Department of Pharmacology, School of Pharmacy, Xinjiang Medical University, Urumqi 830091, China; mdwang963@163.com (M.-D.W.);; 2 Xinjiang Key Laboratory of Biopharmaceuticals and Medical Devices, Urumqi 830001, China; 3 Engineering Research Center of Xinjiang and Central Asian Medicine Resources, Ministry of Education, Urumqi 830017, China; 4 Xinjiang Key Laboratory of Natural Medicines Active Components and Drug Release Technology, Urumqi 830017, China; 5 Charles E. Schmidt College of Science, Florida Atlantic University, Boca Raton, FL 33431, USA; 6 School of Public Health, Xinjiang Medical University, Urumqi 830017, China

**Keywords:** hepatic fibrosis, Periostin, Kupffer cell, single-cell sequencing, drug repurposing

## Abstract

**Background/Objectives**: Liver diseases cause more than 2 million annual deaths globally, accounting for 4% of the total global mortality rate. Hepatic fibrosis (HF) acts as an indispensable pathological mediator in the progressive deterioration of chronic liver diseases. Thus, the identification of effective anti-fibrotic targets and rational development of corresponding therapeutic agents are expected to deliver profound clinical value for patients suffering from chronic liver disorders. **Methods**: An in vivo HF model was established to detect Kupffer cell (KC) polarization and periostin (POSTN) protein expression. In vitro, the CCK-8 (Cell Counting Kit-8) assay was applied to evaluate the regulatory effects of *Postn*-knockdown macrophages on LX-2 cell activity. Conditional knockout mice with *Postn* were constructed in vivo, and liver tissue samples were used for single-cell sequencing. Functional enrichment and cell differentiation prediction analyses were performed. CellChat was further utilized to characterize alterations in intercellular communication between *Postn*-deficient KCs and adjacent liver cells. Finally, POSTN-targeted inhibitors were screened and validated via virtual drug screening and experiments. **Results**: In the HF model, the M2 polarization of KCs was associated with the upregulated expression of POSTN. In contrast, in vitro *Postn* knockdown correlated with significantly suppressed LX-2 cell activation. Single-cell profiling suggests that *Postn* deficiency in Kupffer cells is linked to remodeling of the hepatic microenvironment. In drug repurposing, Rhodiosin exhibited binding affinity to POSTN and was observed to inhibit macrophage M2 polarization. **Conclusions**: POSTN may contribute to KC M2 polarization and be associated with remodeling of the intercellular interaction network among liver cells. Rhodiosin, as a POSTN-binding compound, shows potential for anti-hepatic fibrotic effects.

## 1. Introduction

Liver diseases are responsible for more than 2 million deaths worldwide each year, encompassing cirrhosis, viral hepatitis, and hepatocellular carcinoma, which collectively contribute to 4% of the global mortality burden [1]. Hepatic fibrosis (HF) is a progressive pathological endpoint of chronic liver disorders triggered by viral infections, excessive alcohol consumption, autoimmune diseases, and genetic metabolic disorders. The hallmark pathological manifestations of HF involve hepatocellular injury and aberrant proliferation of hepatic stellate cells (HSCs). Moreover, the activation of hepatic inflammatory responses induced by these etiological factors represents a pivotal risk driver for the initiation and progression of HF. Accumulating evidence has confirmed that hepatocytes, endothelial cells (ECs), and hepatic macrophages initiate the activation of pro-inflammatory signaling pathways—including the transforming growth factor-β (TGF-β) and tumor necrosis factor-α (TNF-α) pathways—under pathological conditions. In response to these inflammatory mediators, predominantly secreted by hepatic macrophages, HSCs undergo phenotypic differentiation into myofibroblasts [2], which leads to excessive extracellular matrix (ECM) deposition and further exacerbates the development and progression of HF [3]. Hepatic macrophages consist of resident Kupffer cells (KCs) that permanently inhabit the hepatic sinusoids, as well as recruited macrophages derived from abdominal tissues and bone marrow monocytes [4]. KCs possess intrinsic self-renewal properties, remain quiescent under physiological conditions with no migratory capacity, and exert indispensable roles in pathogen clearance, phagocytosis of cellular debris, and the regulation of iron metabolism, all of which are critical for the maintenance of hepatic homeostasis. Notably, KC proliferation is a common pathological feature during the progression of HF [5]. Upon liver injury, KCs rapidly secrete interleukin (IL)-1β, TNF-α, and chemokine (C-C motif) ligands 2 and 5, which in turn induce HSCs to secrete ECM components and modulate the recruitment of immune cells to the liver microenvironment [6]. The ECM forms a dense network structure that facilitates leukocyte migration and retention at the injury site [7]. Activated macrophages release pro-fibrotic cytokines to stimulate HSC activation, while activated HSCs sustain the pro-fibrotic phenotype of macrophages by producing the macrophage-stimulating cytokine IL-6 [8]. Emerging studies have demonstrated a reciprocal stimulatory crosstalk between HSCs and KCs: specific ligands drive KCs to secrete TGF-β via an epidermal growth factor receptor-dependent signaling pathway, which plays a central role in the pathogenesis of HF [9].

Periostin (POSTN) is a secreted ECM protein that harbors a conserved structural composition, including an N-terminal emilin domain, four tandem fasciclin domains, a C-terminal heparin-binding domain, and multiple alternatively spliced variants [10]. Furthermore, POSTN mediates fiber cross-linking via direct interactions with core components of the fibrotic matrix, such as fibronectin, collagen I, elastin, and tenascin, which contributes to the formation of a more stable and protease-resistant fibrous network [11]. This structural modification further enhances the maturation and consolidation of fibrotic scar tissue, thereby accelerating the pathological progression of tissue fibrosis.

A tight and bidirectional regulatory interplay exists between POSTN and macrophages. Specifically, POSTN promotes macrophage recruitment and drives their polarization toward the pro-fibrotic M2 phenotype, which in turn exacerbates fibrotic progression [12]. Simultaneously, activated macrophages can upregulate POSTN expression in a positive feedback manner [13]. Mechanistically, POSTN binds to integrin receptors (e.g., αvβ3 and αvβ5) on the cell surface to engage with multiple intracellular signaling cascades [14]. Collectively, POSTN acts as a critical molecular link bridging hepatic inflammation and fibrosis, and serves as a key orchestrator of immune microenvironment regulation during HF. Through a multi-layered “recruitment-polarization-functional modulation” regulatory axis, POSTN cooperates with macrophages to construct a pro-fibrotic liver microenvironment that facilitates the persistent progression of chronic liver disease.

Despite the well-documented critical roles of POSTN in multiple pathological processes including tissue injury, inflammation, and fibrosis, its immunomodulatory functions and underlying mechanisms in the pathogenesis of HF remain largely elusive and under-explored. To address this knowledge gap, the present study systematically elucidated the pro-fibrotic role of POSTN in regulating macrophage polarization and function at single-cell resolution. Additionally, we identified potential anti-fibrotic agents targeting POSTN via virtual drug screening. Collectively, our findings provide novel therapeutic targets and promising candidate drugs to advance the development of effective anti-hepatic fibrosis strategies for clinical translation.

## 2. Results

### 2.1. Increased M2 Polarization of Hepatic Macrophages Correlates with Upregulated POSTN Expression in the HF Model

**Figure 1:**
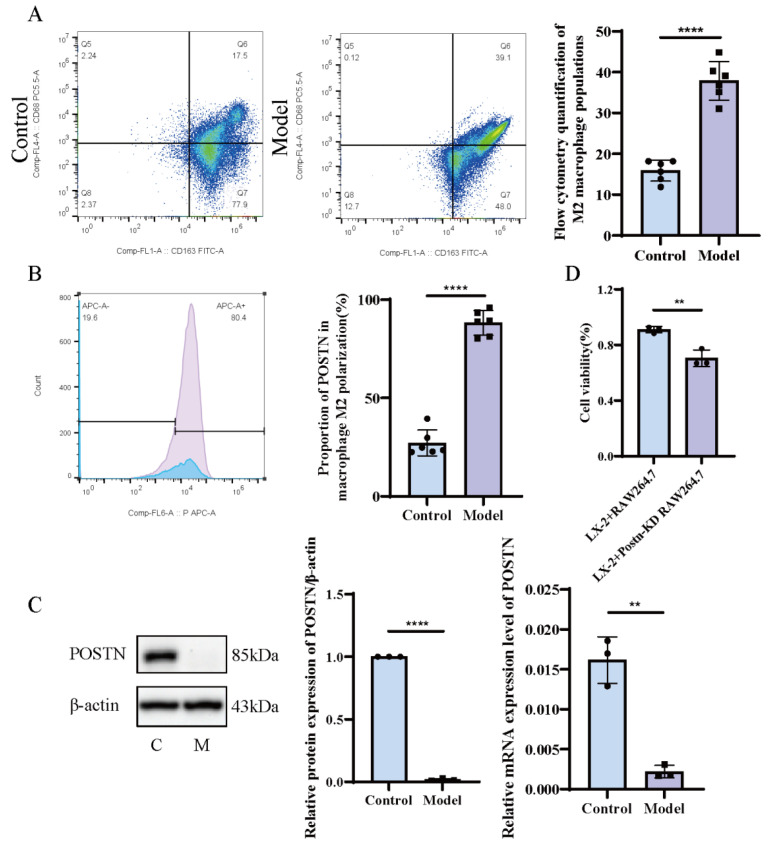
POSTN^+^ macrophages participate in HF by activating HSC. The ratio of M2-type macrophages (CD68^+^CD163^+^) in the HF model of rats changed (**A**), the expression ratio of POSTN in M2-type macrophages (CD68^+^CD163^+^POSTN^+^) changed (Blue: Control group; Purple: Model group) (**B**), the expression verification of macrophage POSTN transfection (**C**), and the influence of macrophages with *Postn* knockdown on the activity of HSC (LX-2) (**D**). ** *p* < 0.01, and **** *p* < 0.0001 vs. model, *n* = 6 (**A**,**B**), *n* = 3 (**C**,**D**) analyzed via Student’s *t*-test.

Hepatic macrophages were isolated from the livers of control and HF model rats, and their polarization status was analyzed by flow cytometry. The proportion of M2-type macrophages (CD163^+^) in the control group was approximately (15.88 ± 2.59)%, whereas that in the model group was significantly elevated to (37.88 ± 4.71)% (Figure 1A). These results are consistent with the possibility that the fibrotic inflammatory microenvironment is associated with the polarization of hepatic macrophages toward the M2 phenotype, and that this may be linked to the pro-fibrotic inflammatory response.

Subsequently, fluorescent antibody labeling was used to detect POSTN protein expression, with fluorescence intensity quantified for semi-quantitative analysis. The proportion of POSTN^+^ cells among M2 macrophages was (27.14 ± 6.60)% in the control group, but increased markedly to (88.27 ± 6.31)% in the model group (Figure 1B). These findings show that POSTN is more highly expressed in M2-type macrophages under fibrotic conditions, which is consistent with a close association between POSTN and M2 polarization of hepatic macrophages during fibrogenesis.

### 2.2. Knockdown of POSTN in Macrophages Inhibits LX-2 Cell Viability

A lentivirus-mediated *Postn*-silenced macrophage cell line was established, and POSTN expression was verified by Western blot and RT-qPCR. Compared with the control group, POSTN protein expression in the *Postn*-knockdown group was (0.018 ± 0.010), showing a significant reduction (*p* < 0.01). Consistently, *Postn* mRNA expression in the knockdown group was (0.0022 ± 0.00079), which was also significantly lower than that in the control group (*p* < 0.01), indicating effective *Postn* silencing (Figure 1C). A macrophage–hepatic stellate cell (HSC) co-culture system was established, and the CCK-8 assay was used to evaluate the effect of *Postn* knockdown on LX-2 cell viability. The results showed that decreased POSTN expression in macrophages was associated with significantly reduced LX-2 cell viability (*p* < 0.01) (Figure 1D).

### 2.3. Quality Control of Hepatic Single-Cell RNA Sequencing (scRNA-Seq) Data

Masson staining of liver tissues from model group mice and detection of the hepatic stellate cell activation marker COL3 revealed that *Postn* knockout significantly alleviated liver pathological changes and fibrosis (Figure S1A) and suppressed COL3 expression (Figure S1B). These findings suggest that *Postn* deficiency may disrupt KC-HSC crosstalk, thus ameliorating liver function.

**Figure 2:**
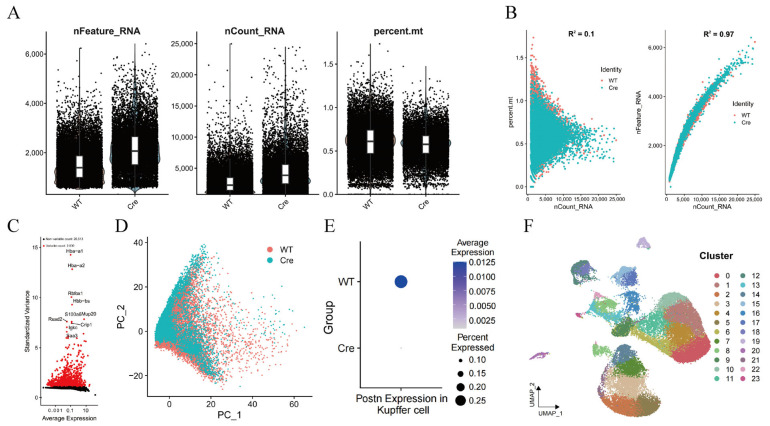
Single-cell data quality control and dimensionality reduction clustering. Detection of gene number, detection count and mitochondrial proportion distribution map (**A**), correlation analysis (**B**), hypervariable gene volcano map (**C**), cell dimension reduction PCA map (**D**), expression of *Postn* in KCs (**E**), cell grouping UMAP (**F**). WT: *Postn^fl/fl^* group; Cre: *Postn^ΔClec4f^* group.

scRNA-seq was performed on liver tissues from KCs-specific *Postn* knockout mice (*Postn^ΔClec4f^*) and control mice (*Postn^fl/fl^*). Quality control analysis revealed that most cells had nFeature_RNA, nCount_RNA, and mitochondrial RNA proportion % (Figure 2A). No linear correlation was observed between nCount_RNA and mitochondrial ratio (R^2^ = 0.1), while a strong positive correlation existed between nCount_RNA and nFeature_RNA (R^2^ = 0.97) (Figure 2B), reflecting rigorous control of sample collection and sequencing processes.

Furthermore, analysis of highly variable genes and principal component analysis (PCA) dimensionality reduction showed significant overlap between the two groups, which is consistent with the absence of batch effects (Figure 2C,D). POSTN expression in KCs was high in the *Postn^fl/fl^* group but barely detectable in the *Postn^ΔClec4f^* group (Figure 2E). UMAP dimensionality reduction identified 24 distinct cell clusters at a resolution of 0.2 (Figure 2F).

### 2.4. KC-Specific Postn Deletion Alters the Transcriptional Profiles of Hepatic Cells

**Figure 3:**
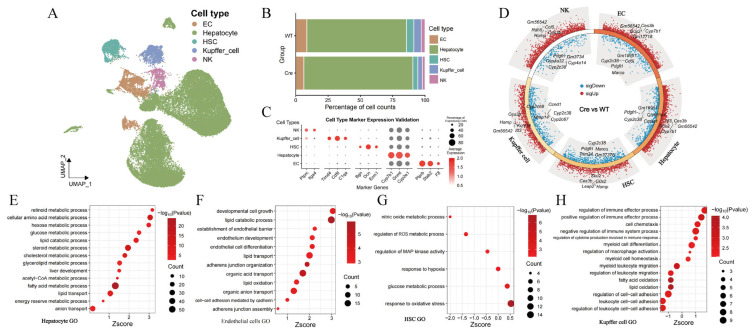
Cell annotation, differential expression and enrichment analysis. Cell type annotation UMAP (**A**), cell proportion analysis (**B**), verification of cell annotation (**C**), differential expression gene analysis (**D**), GO enrichment analysis of hepatocytes (**E**), ECs (**F**), HSC (**G**) and KCs (**H**) in *Postn^ΔClec4f^* group compared with *Postn^fl/fl^* group. WT: *Postn^fl/fl^* group; Cre: *Postn^ΔClec4f^* group.

Cell annotation using marker genes from the SingleR and CellMarker 2.0 databases identified five major hepatic cell types: ECs, hepatocytes, HSCs, KCs, and natural killer (NK) cells (Figure 3A,C). Compared with the *Postn* group, the *Postn^ΔClec4f^* group showed a significant increase in hepatocyte proportion, while the proportions of ECs, KCs, HSCs, and NK cells were markedly decreased (Figure 3B), which points to an association between POSTN and the regulation of KC function as well as their crosstalk with other hepatic cell populations. Differential gene expression analysis revealed substantial transcriptional alterations in all five cell types (Figure 3D). Notably, Cyp7b1, a gene involved in hepatocyte metabolism, was highly expressed in the *Postn^ΔClec4f^* group, which is consistent with enhanced metabolic function. Gene Ontology (GO) enrichment analysis showed significant activation of metabolic pathways in hepatocytes (Figure 3E), while ECs exhibited enhanced differentiation, development, and barrier functions (Figure 3F), observations that correlate with improved EC function following KC-specific *Postn* deletion. In contrast, lower expression levels were observed for genes involved in nitric oxide metabolism, reactive oxygen species (ROS) metabolism, and mitogen-activated protein (MAP) kinase activity in HSCs (Figure 3G). In KCs, immunoregulatory functions were enhanced, whereas certain metabolic and adhesion-related functions were relatively reduced (Figure 3H).

### 2.5. Macrophage POSTN Deficiency Reduces Hepatocyte Inflammation and Enhances Metabolic Capacity

**Figure 4:**
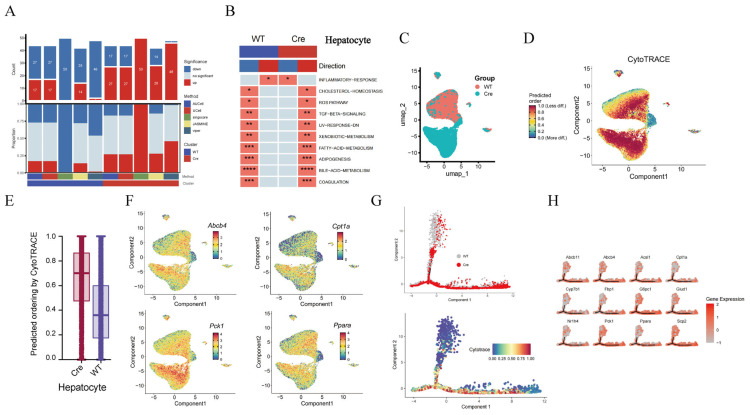
*Postn* deletion of macrophages enhances the metabolic ability of hepatocytes. The number of pathways (**A**) and items (**B**) of hepatocyte GSEA, hepatocyte grouping (**C**), differentiation degree prediction (**D**), differentiation degree score (**E**), hepatocyte metabolism-related gene distribution (**F**), cell differentiation trajectory (**G**) and metabolic gene expression distribution (**H**). WT: *Postn^fl/fl^* group; Cre: *Postn^ΔClec4f^* group. * *p* < 0.05, ** *p* < 0.01, *** *p* < 0.001 and **** *p* < 0.0001.

Gene set enrichment analysis (GSEA) showed that hepatocytes in the *Postn^ΔClec4f^* group had more upregulated than downregulated pathways (Figure 4A), which is consistent with an activated state. The activation ratio was higher in the *Postn^ΔClec4f^* group compared with the *Postn^fl/f^*^l^ group, with reduced hepatic inflammation and activation of all metabolism-related pathways (Figure 4B), raising the possibility of a protective effect of macrophage POSTN deficiency on the liver. Hepatocyte differentiation prediction revealed a lower differentiation degree in the *Postn^ΔClec4f^* group (Figure 4C–E), which was consistent with the differentiation trajectory analysis (Figure 4F). Enhanced hepatocyte metabolism was associated with increased cellular activity, which may be beneficial for maintaining physiological function and ameliorating pathological conditions. For example, genes involved in bile acid metabolism (Abcb4), lipid metabolism (Ppara, Cpt1a), and glucose metabolism (Pck1) were highly expressed in the *Postn^ΔClec4f^* group (Figure 4G,H), suggesting a correlation between macrophage POSTN deficiency and improved liver function, with upregulated hepatocyte metabolic pathways being observed.

### 2.6. Macrophage Postn Deletion Enhances EC Function

**Figure 5:**
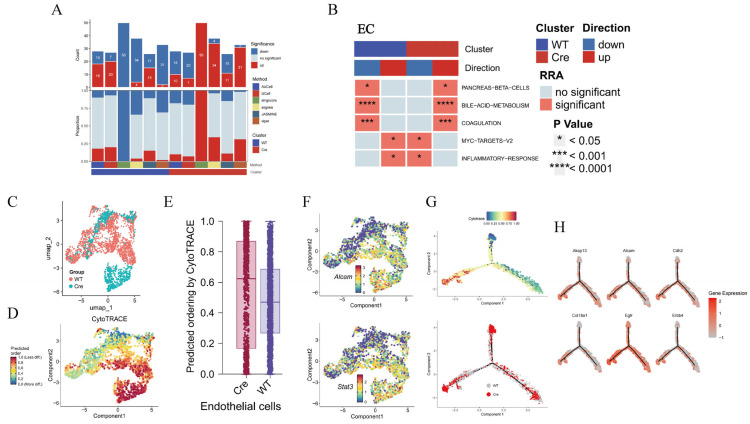
*Postn* deletion of macrophages changes endothelial cell function. The number of pathways (**A**) and items (**B**) of endothelial cell GSEA, endothelial cell grouping (**C**), differentiation degree (**D**), differentiation degree score box diagram (**E**), hepatocyte metabolism-related gene distribution (**F**), cell differentiation trajectory (**G**) and endothelial cell-related gene expression distribution (**H**). WT: *Postn^fl/fl^* group; Cre: *Postn^ΔClec4f^* group.

Compared with the *Postn* group, ECs in the *Postn^ΔClec4f^* group showed overall enhanced function (Figure 5A). Enrichment analysis revealed improved metabolic function and attenuated inflammatory responses (Figure 5B), which is consistent with the possibility that POSTN is associated with the pro-inflammatory effects of macrophages. CytoTRACE results indicated a low differentiation state of ECs in the *Postn^ΔClec4f^* group (Figure 5C–E). Endothelial–mesenchymal transition (EndoMT) is a critical independent driver of HF progression [15]. EC-related genes such as *Stat3* and *Alcam* were highly expressed in the *Postn^ΔClec4f^* group (Figure 5F), suggesting a correlation between macrophage *Postn* deletion and suppression of EndoMT in this context. EC differentiation trajectory analysis showed that the entire *Postn^ΔClec4f^* group localized to the low differentiation region, with elevated expression of endothelial function-related genes (*Cdh2*, *Egfr*, *Alcam*) (Figure 5G,H). These findings are consistent with the notion that macrophage *Postn* deletion is associated with reduced impairment of EC function, which may have implications for HF treatment.

### 2.7. Macrophage POSTN Deficiency Inhibits the Pro-Fibrotic Repair Function of HSCs

**Figure 6:**
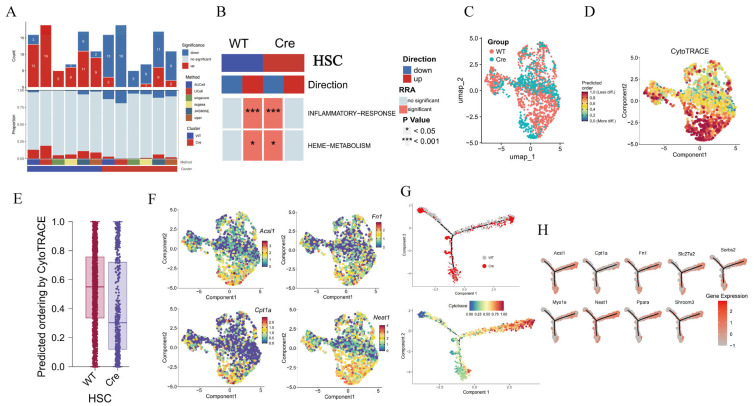
*Postn* deletion of macrophages inhibits the fibrogenic activity of HSC. The number of GSEA pathways (**A**) and items (**B**) of HSC, HSC grouping (**C**), differentiation degree (**D**), differentiation degree score box diagram (**E**), fibrogenic gene distribution (**F**), cell differentiation trajectory (**G**) and endothelial cell-related gene expression distribution (**H**). WT: *Postn^fl/fl^* group; Cre: *Postn^ΔClec4f^* group.

GSEA analysis revealed that most HSC-related pathways were upregulated in the *Postn^fl/fl^* group, while the number of activated pathways was significantly reduced in the *Postn^ΔClec4f^* group (Figure 6A), particularly pathways related to inflammation and metabolism (Figure 6B). This is consistent with marked suppression of HSC overactivation, which may be associated with alleviated HSC hyperactivity in HF. Based on differentiation predictions, the *Postn^ΔClec4f^* group exhibited a highly differentiated state (Figure 6C–E), with low expression of HSC activation-related genes (Acsl1, Fn1, Cpt1a, Neat1) (Figure 6F). In contrast, the *Postn^fl/fl^* group predominantly localized to the right section of the differentiation trajectory (Figure 6G), with high expression of HSC fibrosis-related genes (Figure 6H). These results suggest a correlation between macrophage POSTN deficiency and reduced pro-fibrotic repair function of HSCs, raising the possibility that this association may influence HF progression.

### 2.8. The Loss of Postn in Macrophages Is Accompanied by a Decrease in M2 Polarization Markers

**Figure 7:**
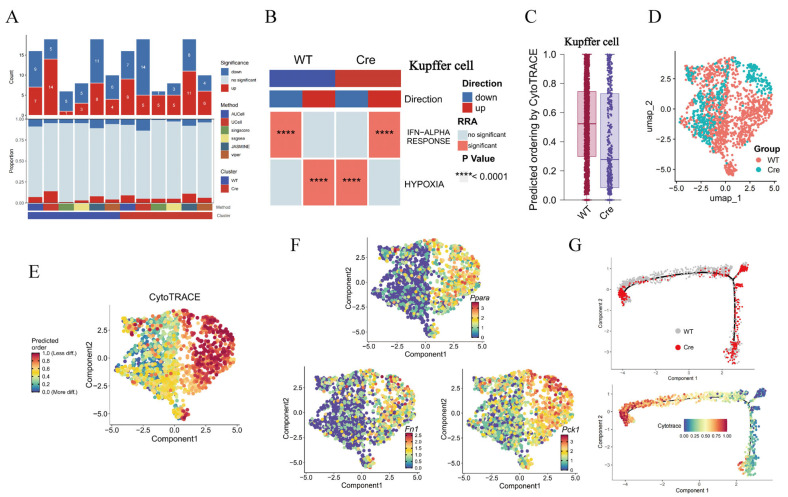
*Postn* deletion inhibits M2 polarization of macrophages. The number (**A**) and item (**B**) of GSEA in KCs, KC grouping (**C**), differentiation degree (**D**), differentiation degree score box diagram (**E**), M2 polarized gene distribution (**F**), and cell differentiation trajectory (**G**). WT: *Postn^fl/fl^* group; Cre: *Postn^ΔClec4f^* group.

GSEA analysis showed that *Postn* deletion had little effect on the number of macrophage-enriched pathways (Figure 7A) but was associated with reduced hypoxia and IFN-α activation (Figure 7B). Hypoxia is crucial for tissue repair and is closely associated with fibrosis, particularly tissue hypoxia. This raises the possibility that *Postn* deletion may be linked to impaired HF progression, potentially through an association with reduced macrophage-mediated hypoxia responses. Differentiation prediction revealed that the *Postn^ΔClec4f^* group was in a highly differentiated state, whereas the *Postn^fl/fl^* group was poorly differentiated (Figure 7C–E). In the *Postn^ΔClec4f^* group, M2 polarization markers (Ppara, Pck1) and the fibrogenic molecule Fn1 were all lowly expressed (Figure 7F). Consistent with the differentiation degree, the *Postn^ΔClec4f^* group localized to the highly differentiated region of the trajectory (Figure 7G). Collectively, these findings are consistent with the possibility that *Postn* deletion is associated with reduced macrophage M2 polarization and pro-fibrotic activity, and that this association may involve attenuated hypoxic responses.

### 2.9. Postn Deletion of Macrophages Changes the Pattern of Intercellular Interaction

**Figure 8:**
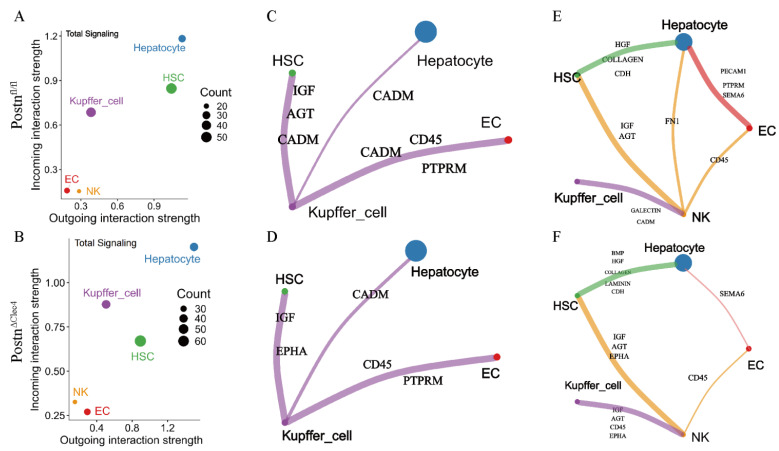
*Postn* deletion changes the interaction pattern of liver cells. Cell communication intensity (**A**,**B**), direct effect (**C**,**D**) and indirect effect (**E**,**F**) of KCs on other cells in the two groups. WT: *Postn^fl/fl^* group; Cre: *Postn^ΔClec4f^* group.

To investigate how POSTN may influence hepatic cell functions, intercellular communication analysis was performed to examine the interaction patterns between KCs and other hepatic cell types. The signal role scatter plot showed increased output intensity of hepatocytes in the *Postn^ΔClec4f^* group, while both output and input intensities of HSCs were decreased (Figure 8A,B), which is consistent with enhanced hepatocyte activity and diminished HSC function. Regarding specific signaling pathways, the number of output signals from KCs to HSCs appeared reduced in the *Postn^ΔClec4f^* group compared with the *Postn^fl/fl^* group, and the signal composition was altered (Figure 8C,D). Notably, the AGT signal, which is involved in tissue repair [16], was not detected. Concurrently, the influence of KCs on ECs also appeared diminished, as evidenced by reduced CADM signaling (associated with cell adhesion) [17]. Since KCs showed no direct signaling differences from hepatocytes, we hypothesized that NK cells might mediate indirect crosstalk. The results showed that NK cells in the *Postn^ΔClec4f^* group had a reduced effect on hepatocyte Fn1 release compared with the *Postn^fl/fl^* group (Figure 8E,F). Meanwhile, the indirect influence of NK cells on hepatocytes via HSCs was found to be amplified, raising the possibility that macrophage POSTN deficiency may be associated with altered hepatocyte metabolism through NK cells. Collectively, macrophage *Postn* deletion is associated with multiple alterations in hepatic cell functions, which may be linked to remodeled intercellular communication patterns.

### 2.10. Rhodiosin May Be a Candidate Inhibitor for Targeting POSTN

**Figure 9:**
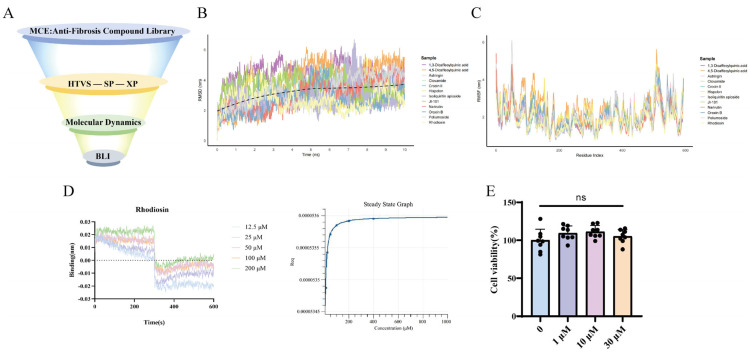
Rhodiosin was screened as a candidate inhibitor of POSTN protein. Screening process of targeted molecules (**A**), binding stability of 12 candidate inhibitors with POSTN (**B**) and its effect on the α-helix structure of the target protein (**C**), detection of binding affinity of Rhodiosin with POSTN protein by BLI (**D**), and effect of Rhodiosin on the cell viability of RAW264.7 (**E**). ^ns^ *p* > 0.05, vs. 1 μM, 10 μM, and 30 μM, *n* = 8, analyzed via one-way analysis of variance (ANOVA).

Given the pro-fibrotic role of macrophage-derived POSTN, targeted inhibitors were screened as novel therapeutic strategies for HF. A total of 1922 potential anti-fibrotic drug molecules were retrieved from the MCE database. Through hierarchical molecular docking (HTVS, SP, and XP), 49 candidate POSTN inhibitors were suggested (Figure 9A). Among the compounds with favorable docking scores, Crocin II exhibited superior binding energy compared to Rhodiosin, whereas Rhodiosin showed slightly better ligand efficiency and 10 ns MD stability (RMSD ≈ 2.62 Å) (Table S1). All candidate compounds stably bound to POSTN and induced α-helical changes in the protein (Figure 9B,C). Biolayer interferometry (BLI) experiments showed a high binding affinity between Rhodiosin and POSTN (KD = 2.605 × 10^−8^ M, Figure 9D); Rhodiosin formed stable and specific binding with the target protein over the 100 ns simulation, primarily maintained by hydrogen bonds and hydrophobic interactions, without ligand detachment or protein structural destabilization, providing a reliable structural basis for subsequent functional experiments. To further improve screening accuracy and mitigate the limitations of docking scoring functions, the binding free energy of the candidate complex was calculated using the Prime MM-GBSA module. The calculation was performed with the VSGB solvent model and the OPLS_2005 force field, allowing flexible optimization of amino acid side chains within 5.0 Å of the ligand. The calculated binding free energy was −62.54 kcal/mol, suggesting that Rhodiosin may be a potential POSTN-targeted inhibitor. In vitro toxicity assays showed that Rhodiosin had not significant effect on macrophage viability at concentrations of 1 μM, 10 μM, and 30 μM (Figure 9E), implying low cytotoxicity.

### 2.11. Rhodiosin Inhibits POSTN and M2 Polarization of Macrophages

**Figure 10:**
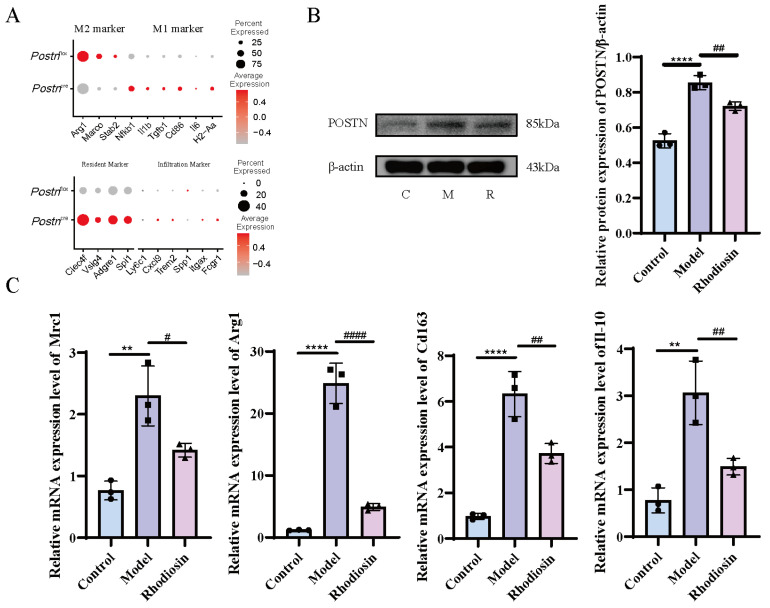
Therapeutic potential of Rhodiosin for HF. *Postn*’s M1/M2 polarization markers and residency/infiltration markers (**A**), effect of Rhodiosin on POSTN protein expression in the macrophage M2 polarization model (**B**), effect of Rhodiosin on mRNA expression of M2 polarization markers (**C**). ** *p* < 0.01, and **** *p* < 0.0001 vs. model, *n* = 3, analyzed via Student’s *t*-test. ^#^ *p* < 0.05, ^##^ *p* < 0.01, and ^####^ *p* < 0.0001 vs. Rhodiosin, *n* = 3, analyzed via Student’s *t*-test.

Analysis of cell polarization markers revealed that, as expected, Postn knockout decreased classical M2 marker expression while increasing M1 marker expression. Furthermore, Kupffer cells (KCs) in the liver serve as a major reservoir of human macrophages, suggesting they are predominantly resident-derived rather than infiltrating. Marker analysis showed that KCs in both groups expressed high levels of resident markers and low levels of infiltrating markers (Figure 10A).

To elucidate the therapeutic potential of Rhodiosin for HF, an M2 polarization model was established, and the expression of POSTN protein along with the M2 polarization phenotype of macrophages was assessed using Western blot analysis. The results indicated that Rhodiosin significantly inhibited POSTN protein expression (*p* < 0.05, Figure 10B). Additionally, qPCR analysis revealed that the mRNA levels of M2 polarized markers, including Mrc1, Arg1, Cd163, and Il-10, were significantly downregulated in the Rhodiosin group compared to the model group (Figure 10C). Using protein–protein docking, we found that Rhodiosin leads to a general increase in the docking scores (i.e., a decrease in absolute energy values) of the POSTN complexes with ITGAV, ITGB4, and ITGB5, as well as a decrease in confidence scores. This suggests that it may effectively inhibit/attenuate the binding of POSTN to downstream integrins by inducing steric hindrance or causing conformational changes in POSTN (Figure S4).

qPCR results showed that M2 polarization promoted the mRNA expression of α-SMA, COL1A1, COL3A1, and MMP2 in fibroblasts, and Rhodiosin significantly reversed these effects (Figure S5).

## 3. Discussion

The pathogenesis of HF is centered on the activation of HSCs and the subsequent excessive deposition of ECM [18,19]. This pathological accumulation of ECM disrupts liver architecture and impairs hepatic function, highlighting the inhibition of HSC activation as a critical therapeutic strategy for HF prevention and treatment. Following liver injury, hepatic macrophages are activated and secrete a spectrum of pro-inflammatory cytokines [20], which drive HSC activation and amplify the fibrotic cascade [21,22,23]. In turn, activated HSCs release pro-inflammatory factors and ECM components that recruit additional macrophages, forming a pro-fibrotic positive feedback loop that sustains disease progression [24]. Accumulating evidence indicates that the fibrogenic potential of macrophages is closely linked to M2 polarization and the upregulation of pro-fibrotic mediators [25,26]. POSTN, a pro-fibrotic molecule involved in cell adhesion and migration, has recently been implicated in fibroblast-mediated fibrosis across multiple tissues [27,28]. Consistent with these findings, the present study observed a marked expansion of reparative M2-type macrophages in HF, accompanied by elevated POSTN expression. Furthermore, *Postn* knockdown in macrophages was associated with significantly inhibited HSC activity, suggesting a pivotal role of POSTN in mediating crosstalk between KCs and HSCs during fibrogenesis.

To delineate the specific role of POSTN in hepatic pathophysiology, we generated *Postn^fl/fl^* mice and KCs-specific *Postn* conditional knockout mice (*Postn^ΔClec4f^*). Leveraging the high resolution and comprehensiveness of scRNA-seq, we systematically characterized the pro-fibrotic features associated with KC-derived POSTN at the single-cell level. KC-specific *Postn* deletion induced profound alterations in hepatic cell composition and function, including enhanced hepatocyte metabolic activity, attenuated endothelial cell (EC) inflammation, impaired HSC pro-fibrotic function, and reduced KC M2 polarization and fibrogenic potential. Given that hepatocyte metabolism is a key determinant of hepatic function, our findings raise the possibility that macrophage POSTN deficiency mitigates pro-inflammatory responses, potentially through indirect regulation by natural killer (NK) cells. In contrast to hepatocytes, the attenuation of EC inflammation following *Postn* deletion may be associated with reduced macrophage CADM signaling, with no direct involvement of NK cells. Angiotensinogen (AGT), a liver-derived molecule involved in blood pressure regulation and tissue repair, was found to be downregulated in *Postn^ΔClec4f^* mice; thus, KC POSTN deletion may contribute to inhibited HSC activation by reducing AGT signaling. For KCs themselves, POSTN is primarily linked to mediating hypoxia responses, cell polarization, and fibrogenic promotion. Collectively, these data suggest that KC-specific *Postn* conditional knockout reestablishes the homeostatic balance of hepatic cellular states, creating a microenvironment characterized by enhanced metabolic activity and diminished inflammatory and fibrotic propensity—findings that point to POSTN as a potential therapeutic target for HF.

Given the current lack of POSTN-targeted inhibitors, we employed a stepwise screening strategy to search for small-molecule targeted drugs. Through hierarchical molecular docking and molecular dynamics simulations, 12 candidate compounds (including Rhodiosin) were identified as potential POSTN inhibitors. Rhodiosin, a natural compound derived primarily from Rhodiola species, exhibits well-documented antioxidative, neuroprotective, and anti-inflammatory properties [29]. Combined with BLI assays and in vitro functional validation, we observed that Rhodiosin targets POSTN and correlates with inhibited macrophage M2 polarization. Notably, Rhodiosin exhibited low cytotoxicity at therapeutically relevant concentrations, suggesting its potential as an anti-fibrotic agent. Nevertheless, previous studies have demonstrated the protective effects of Rhodiosin against drug-induced hepatotoxicity [30], further supporting its translational potential for HF treatment. However, overexploitation of Rhodiola sachalinensis has led to a significant increase in extraction costs, limiting further in vivo evaluation of its anti-fibrotic efficacy. At present, the production of Rhodiosin relies primarily on extraction and isolation from wild plants. Using a new biosynthesis platform for flavonoid glycosides [31], a microbial synthesis process for rhodiosin can be established from glucose as the raw material. through heterologous expression of the glycosyltransferases required for Rhodiosin biosynthesis (e.g., the UDP-glycosyltransferase responsible for the glycosylation of herbacetin to Rhodiosin) in *Escherichia coli* or yeast.

## 4. Materials and Methods

### 4.1. Reagents

Carbon tetrachloride (CCl_4_, Cat. No. C805325) was purchased from Macklin Biochemical Co., Ltd. (Shanghai, China). APC/Cyanine7 anti-mouse CD45 (Cat. No. 157618), Alexa Fluor 700 anti-mouse CD86 (Cat. No. 159214), and purified anti-mouse CD163 recombinant antibody (Cat. No. 11802) for flow cytometry were obtained from BioLegend (San Diego, CA, USA). Dulbecco’s modified Eagle’s medium (DMEM) and fetal bovine serum (FBS) were supplied by Gibco (Grand Island, NY, USA). All lentiviral vectors were synthesized by Jikai Gene Technology Co., Ltd. (Shanghai, China). Cell Counting Kit-8 (CCK-8) was purchased from Bioss Biotechnology Co., Ltd. (Beijing, China). Interleukin-4 (IL-4, Cat. No. 062349) was obtained from PeproTech (Cranbury, NJ, USA). Rhodiosin (Cat. No. HY-N2425) was purchased from MedChemExpress (MCE, Monmouth Junction, NJ, USA). Primers for reverse transcription–quantitative polymerase chain reaction (RT-qPCR) were synthesized by Sangon Biotech (Shanghai) Co., Ltd. (Shanghai, China). Anti-POSTN antibody and anti-β-actin antibody were acquired from Proteintech Group, Inc. (Wuhan, China).

### 4.2. Cell Culture

RAW264.7 murine mononuclear macrophage leukemia cells (Cat. No. CL-0190) and LX-2 human hepatic stellate cells (Cat. No. CL-0560) were both purchased from Wuhan Procell Life Science & Technology Co., Ltd. (Wuhan, China). Cells were cultured in DMEM supplemented with 10% FBS, 100 U/mL penicillin and 100 μg/mL streptomycin. All cultures were maintained in a humidified incubator at 37 °C with 5% CO_2_.

### 4.3. Generation of KC-Specific Postn Conditional Knockout Mice

Clec4f-iCre mice were constructed via gene-editing technology, with an internal ribosome entry site (IRES)-iCre cassette inserted into the 3′ untranslated region (3′UTR) of the Clec4f gene. Crossbreeding with loxP-flanked mice enabled Cre recombinase-mediated sequence recombination between loxP sites specifically in KCs. Clec4f-iCre mice (Stock No. C001353) and *Postn^fl/fl^* mice (Stock No. S-CKO-11368) were purchased from Cyagen Biosciences (Guangzhou, China). KC-specific *Postn* knockout mice (*Postn^ΔClec4f^*) were generated by crossing *Postn^fl/fl^* mice with Clec4f-iCre transgenic mice.

### 4.4. The Establishment of the Hepatic Fibrosis Model

SD rats were provided by the Animal Experimental Center of Xinjiang Medical University, and *Postn^ΔClec4f^* mice were obtained from Cyagen Biosciences (Guangzhou, China). All male rats were housed under standard laboratory conditions, while mice (half male and half female) were maintained in a specific pathogen-free (SPF) environment with free access to standard rodent chow and sterile water. Rats and mice were randomly divided into two groups (n = 10 per group): the control group and the CCl_4_-induced HF model group. Mice and rats received intraperitoneal injections of olive oil or 20% CCl_4_ in olive oil twice weekly for 6 consecutive weeks. All animal experiments were approved by the Animal Ethics Committee of Xinjiang Medical University (IAUC-20241008-50).

### 4.5. Flow Cytometry Analysis of Hepatic Macrophage Polarization in Rats

Rats were anesthetized via intraperitoneal injection of sodium pentobarbital and fixed on a sterile operating table. After exposing the hepatic portal vein, liver perfusion was performed, followed by liver dissection, enzymatic digestion and filtration to isolate parenchymal cells. Rat KCs were purified by Percoll density gradient centrifugation, and cell density was adjusted to 1 × 10^6^ cells/mL. Cells were stained with anti-CD68, anti-CD86 and anti-CD206 antibodies for 45 min at 4 °C in the dark, then washed twice with 1 mL phosphate-buffered saline (PBS). Cells were resuspended in 300 μL PBS and subjected to flow cytometry analysis on a BD flow cytometer (BD Biosciences, San Jose, CA, USA).

### 4.6. Tissue Processing and Single-Cell RNA Sequencing

Fresh liver tissues were rinsed with cold PBS and cut into 0.5 mm^2^ fragments, followed by repeated washing, mincing, enzymatic dissociation and red blood cell lysis. Cell viability was determined by trypan blue staining using a Luna-FL Fluorescent Cell Counter. Single-cell libraries were constructed with the 10× Genomics Chromium Single-Cell 3′ Reagent Kit (10x Genomics, Shanghai, China), and paired-end sequencing (150 bp) was performed on an Illumina sequencing platform. Given the lack of a stable KC cell line, the RAW264.7 macrophage cell line was used as an in vitro cell model for subsequent experiments.

### 4.7. Quality Control of Single-Cell Data

Seurat software (v5.2.0) was used for single-cell data quality control, with filtering criteria including the number of expressed genes per cell (nFeature_RNA), unique molecular identifier (UMI) count (nCount_RNA) and mitochondrial gene ratio (percent.mt). Cells with nFeature_RNA > 6000 or <500 and nCount_RNA > 15,000 or <500 were excluded. After quality control, the gene expression matrix was log-normalized and subjected to feature selection, with highly variable genes retained for downstream analyses. Cell annotation was performed using a strategy of reference database matching + marker gene validation: SingleR software(v2.12.0) was applied for initial cell type assignment by matching quality-controlled cells to the Human Primary Cell Atlas reference dataset; accurate cell type annotation was further confirmed using the CellMarker 2.0 database for subsequent analysis.

### 4.8. Differential Expression Gene Analysis and Enrichment Analysis

Based on well-annotated cell clusters, DEGs between groups of the same cell type were identified using the Seurat FindMarkers function with the Wilcoxon rank-sum test, with the cutoff criteria of adjusted *p* value (*p*.adj) < 0.05 and |log_2_fold change (FC)| ≥ 1. Gene Ontology (GO) biological process (BP) and Kyoto Encyclopedia of Genes and Genomes (KEGG) pathway enrichment analyses were conducted using the clusterProfiler R package, with significant enrichment defined as *p*.adj < 0.05. Gene set enrichment analysis (GSEA) was performed via the irGSEA R package against the hallmark gene sets from the Molecular Signatures Database (MSigDB), with false discovery rate (FDR) < 0.05 as the threshold for significant enrichment, to elucidate core biological functions and signaling pathways of DEGs.

### 4.9. Cell Differentiation Potential Prediction, Cell Trajectory and Cell-to-Cell Communication Analysis

CytoTRACE(v0.3.3) was used to predict the differentiation potential of cells within the same type, with CytoTRACE scores calculated based on single-cell transcriptomic gene expression complexity (higher scores indicating stronger differentiation potential). Differences in differentiation capacity among cell clusters were visualized by expression heatmaps. Single-cell trajectory analysis was performed using Monocle2: with annotated cell clusters as input, pseudo-time developmental trajectories were constructed via gene filtering, dimensionality reduction (DDRTree algorithm) and cell ordering to define cell differentiation paths and key branch nodes and reveal the dynamic differentiation process of cell populations. Intercellular communication was analyzed using the CellChat(v2.2.0) R package: a cell–ligand–receptor interaction network was constructed based on cell type annotations, and biologically significant ligand–receptor pairs were screened against the built-in database. Communication probability was calculated to quantify intercellular interaction intensity and characterize cell–cell regulatory relationships.

### 4.10. Lentivirus-Mediated Postn Silencing in RAW264.7 Cells

Lentiviral packaging cells were co-transfected with the constructed lentiviral vector and packaging mix for viral packaging. Viral stock was collected, concentrated by ultrafiltration and titrated (gene sequences are shown in Table 1). Experiments were divided into two groups: the control group was treated with an equal volume of complete medium (without transfection reagent) to maintain basal *Postn* expression; the model group was transfected with *Postn*-targeting shRNA to specifically knock down *Postn* expression. RAW264.7 cells in the logarithmic growth phase were seeded into 6-well plates at a density of 5 × 10^5^ cells per well 24 h prior to transfection. When cell confluence reached 80%, lentivirus was diluted in medium to a multiplicity of infection (MOI) of 150 for transfection. After 24 h of transfection, the medium was replaced with fresh complete medium for further culture.

### 4.11. CCK-8 Assay for LX-2 Cell Viability in LX-2/Macrophage Co-Culture System

The CCK-8 assay was performed to evaluate the proliferative activity of LX-2 cells in the LX-2/macrophage co-culture system. LX-2 cells co-cultured with negative control-transfected RAW264.7 cells served as the control group, and LX-2 cells co-cultured with *Postn*-transfected RAW264.7 cells were assigned as the model group. When LX-2 and RAW264.7 cells reached 80% confluence, cells were subcultured in Transwell chambers, with LX-2 cells seeded in the lower chamber at a density of 1 × 10^5^ cells/well and RAW264.7 cells in the upper chamber at 1 × 10^4^ cells/well. After 24 h of co-culture, CCK-8 reagent was added, and the optical density (OD) values were measured subsequently.

### 4.12. Protein–Protein Docking

To predict and evaluate the near-native three-dimensional structures of the complexes and the effect of Rhodiosin on protein–protein interactions, protein–protein molecular docking simulations were performed using the HDOCKlite v1.1 local server. The receptor/ligand structures included the POSTN monomer, the POSTN–Rhodiosin complex, and three integrin proteins: ITGAV (PDB ID: 1JV2, chain A), ITGB4 (AlphaFold-predicted structure), and ITGB5 (PDB ID: 1QG3, chain A). HDOCK employs a hybrid algorithm combining physicochemical principles and bioinformatics to develop an efficient docking algorithm and an accurate scoring function. Since no prior binding site information for the protein complexes was available, an ab initio global docking strategy was adopted, which performs a thorough sampling of the six degrees of translational and rotational freedom of the two molecules in three-dimensional space to explore all possible putative binding modes. The generated binding conformations were then ranked and evaluated using the HDOCK-built scoring function. Docking results were assessed using two core metrics: (1) Docking score—based on energy evaluation; a more negative score indicates lower system energy and greater stability of the binding model. (2) Confidence score—used to evaluate the probability of interaction between the two molecules. A confidence score > 0.7 indicates a high likelihood of binding; between 0.5 and 0.7 suggests possible binding; below 0.5 indicates a low binding probability.

### 4.13. Virtual Screening and Molecular Dynamics Simulation

The crystal structure of POSTN (PDB ID: 5YJG) was retrieved from the Protein Data Bank (PDB), and subjected to structural optimization using the Protein Preparation Wizard module in Schrödinger software (v2024-1), including correction of structural defects, completion of missing side chains and hydrogen bond optimization. Ligand molecules from the Traditional Chinese Medicine Systems Pharmacology Database and Analysis Platform (TCMSP) and Food and Drug Administration (FDA) database were processed with LigPrep, involving hydrogenation, tautomer generation, desalting, and ionization state calculation under the OPLS4 force field; multiple stereoisomers were generated for each ligand [32]. A receptor grid of the specific POSTN-binding domain was constructed using the receptor grid generation module in Schrödinger. In this study, the SiteMap module was used to analyze the surface topology of the protein to identify potential drug-binding sites. The detection logic was based on pocket enclosure, cavity volume, and physicochemical properties. According to the SiteMap results, the cavity exhibiting the optimal binding volume and enclosure (Site Score = 1.017) was selected as the target pocket for virtual screening. Subsequently, using the centroid of this predicted site as the coordinate center, a grid was generated with the Glide Grid Generation tool(v2024-1). The inner box dimensions were set to 30 Å × 20 Å × 20 Å (X: 34.75, Y: 1.74, Z: 183.95) to ensure coverage of the entire candidate binding region. Hierarchical virtual screening was performed via Glide with three sequential steps—high-throughput virtual screening (HTVS), standard precision (SP) screening and extra precision (XP) screening—with multiple conformations output for each ligand. Molecular dynamics simulations were conducted using the Desmond module in Schrödinger [33], including energy minimization, heating to 300 K, 10 ns NPT equilibration, and treatment of non-bonded interactions via the particle mesh Ewald (PME) method.

### 4.14. Effect of Postn Inhibitor Rhodiosin on Macrophage M2 Model

An IL-4-induced M2 macrophage polarization model was established with 20 ng/mL IL-4, followed by treatment with 30 μM Rhodiosin (a POSTN-specific inhibitor) for 24 h. RAW264.7 cells were harvested from 6-well plates, and total cellular proteins were extracted by adding protein lysis buffer, with protein concentration quantified using a BCA protein assay kit. A total of 20 μL protein lysate was subjected to sodium dodecyl sulfate–polyacrylamide gel electrophoresis (SDS-PAGE), and separated proteins were transferred onto polyvinylidene fluoride (PVDF) membranes. Membranes were blocked at room temperature for 2 h, then incubated with the corresponding primary antibodies at 4 °C overnight. After washing, membranes were incubated with secondary antibodies at room temperature for 2 h, and protein bands were visualized using a chemiluminescence imaging system. The gray values of POSTN and reference proteins were quantified and analyzed with ImageJ software(1.54g).

Total cellular RNA was extracted using the TRIzol reagent, and reverse transcription was performed to synthesize cDNA. Quantitative real-time PCR (qPCR) amplification was conducted under the following cycling conditions: initial denaturation at 94 °C for 5 min; followed by 30 cycles of denaturation at 94 °C for 30 s, annealing at 58 °C for 30 s, and extension at 72 °C for 30 s; with a final extension at 72 °C for 10 min and holding at 4 °C for 10 min. Glyceraldehyde-3-phosphate dehydrogenase (GAPDH) was used as the internal reference gene, and the relative mRNA expression levels were calculated using the 2^−ΔΔCt^ method. Primer sequences are listed in Table 2.

### 4.15. Statistical Treatment

All statistical analyses were performed using GraphPad Prism 9 software, and experimental data were presented as the mean ± standard deviation (SD). Comparisons between two groups were analyzed using the unpaired Student’s *t*-test, and comparisons among multiple groups were conducted via one-way analysis of variance (ANOVA).

## 5. Conclusions

In conclusion, the present study, utilizing *Postn* knockout mouse models, scRNA-seq, and drug molecular screening, is the first to systematically explore the association of POSTN^+^ macrophages with HF pathogenesis. We suggest a potential mechanism by which POSTN may be involved in macrophage M2 polarization that correlates with fibrosis, and identified Rhodiosin as a novel POSTN-associated inhibitor through virtual screening, thereby proposing a new therapeutic target and candidate drug for HF treatment. Despite these contributions, this study has several limitations. First, while the CCl_4_-induced HF model effectively recapitulates the core pathological features of the disease, species-specific differences in the immune microenvironment and transcriptional regulatory networks between animals and humans necessitate further validation of our findings using human organoid models or clinical samples. Second, the long-term safety, pharmacokinetic profiles, and metabolic stability of Rhodiosin—as a potential POSTN-targeted small-molecule inhibitor—require comprehensive evaluation through systematic preclinical studies and subsequent clinical trials to support its translational application.

## Figures and Tables

**Table 1:** Gene sequence of *postn* knockdown.

Oligo Name	Top Strand (5′-3′)	Bottom Strand (5′-3′)
ZF-KD-31004-A	GATCCGGAGAACAATGTCAATGTTTTCAAGAGAAACATTGACATTGTTCTCCTTTTTTG	AATTCAAAAAAGGAGAACAATGTCAATGTTTCTCTTGAAAACATTGACATTGTTCTCCG
ZF-KD-31004-B	GATCCGCATGGTTATTCCTTCAATTTCAAGAGAATTGAAGGAATAACCATGCTTTTTTG	AATTCAAAAAAGCATGGTTATTCCTTCAATTCTCTTGAAATTGAAGGAATAACCATGCG
ZF-KD-31004-C	GATCCGGAACCAGATTGCCACAAATTCAAGAGATTTGTGGCAATCTGGTTCCTTTTTTG	AATTCAAAAAAGGAACCAGATTGCCACAAATCTCTTGAATTTGTGGCAATCTGGTTCCG

**Table 2:** Primer sequences.

Primers	Forward Sequence (5′-3′)	Reverse Sequence (5′-3′)
*GAPDH*	AACTCCCACTCTTCCACCTTCG	TCCACCACCCTGTTGCTGTAG
*Arg1*	ACATTGGCTTGCGAGACGTA	ATCACCTTGCCAATCCCCAG
*Mrc1*	AAATGGCTTCCTGGAGAGCC	ACCCTCCGGTACTACAGCAT
*Cd163*	GAGAAGACGCTGGTGTGACA	CCAAGCTGTCTGCAAACCAC
*IL10*	CAGAGAAGCATGGCCCAGAA	GCTCCACTGCCTTGCTCTTA

## Data Availability

The original contributions presented in this study are included in the article/Supplementary Material. Further inquiries can be directed to the corresponding authors.

## Supplementary Materials

The following supporting information can be downloaded at https://www.mdpi.com/article/10.3390/ph19050752/s1: Figure S1: Postn knockdown attenuates the severity of liver injury in mice. Postn knockdown significantly alleviates histopathological changes and collagen deposition in liver tissue (A). POSTN knockdown markedly reduces the expression level of COL3 (B). *** *p* < 0.001, and **** *p* < 0.0001 vs. *Postn^ΔClec4f^* group, *n* = 4, analyzed via Student’s *t*-test; Table S1: MM-GBSA binding free energies (ΔG bind) and ligand efficiencies of candidate compounds against POSTN protein; Figure S2: RMSD, RMSF, protein–ligand contacts and ligand–protein contacts; Figure S3: Schematic diagram of receptor–ligand interactions for all key candidate molecules; Figure S4: Rhodiosin may exert its effects by blocking the binding of POSTN to integrins; Figure S5: Rhodiosin could contribute to the inhibition of HSC activation and fibrosis formation. * *p* < 0.05, ** *p* < 0.01, and *** *p* < 0.001 vs. model, ^#^ *p* < 0.05, ^##^ *p* < 0.01, and ^###^ *p* < 0.001 vs. Rhodiosin, *n* = 4, analyzed via one-way analysis of variance (ANOVA).
