# Supplementary material for: Molecular Mechanism of POSTN Mediating M2 Polarization of Kupffer Cells to Promote Hepatic Fibrosis
Source: Pharmaceuticals (Basel). 2026 May 11;19(5):752. doi: 10.3390/ph19050752 (PMC13210759; doi:10.3390/ph19050752)
Supplement: Supplementary file 1 [file pharmaceuticals-19-00752-s001.zip › pharmaceuticals-4246614-supplementary.pdf]

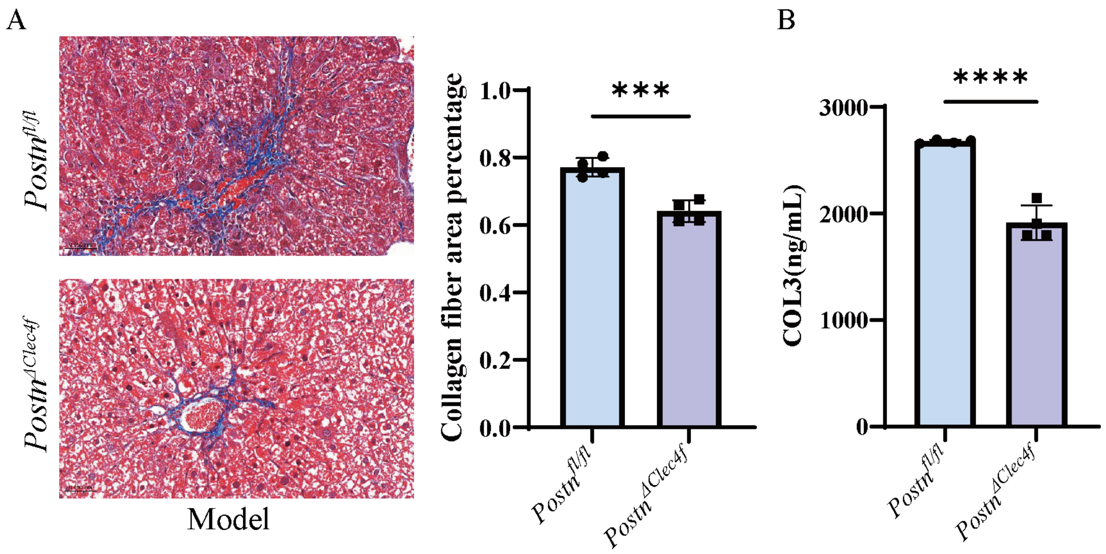

Figure S1. Postn knockdown attenuates the severity of liver injury in mice. Postn knockdown significantly alleviates histopathological changes and collagen deposition in liver tissue (A). POSTN knockdown markedly reduces the expression level of COL3 (B). ***P < 0.001, and ****P < 0.0001 *vs.* *Postn*$^{\Delta Clec4f}$ group, n=4, analyzed via Student's *t*-test.

Table S1. MM-GBSA binding free energies (ΔG bind) and ligand efficiencies of candidate compounds against POSTN protein.

Compound Name	MMGBSA dG Bind (kcal/mol)	MMGBSA dG Bind(NS)	Prime MMGBSA Ligand Efficiency
Crocin II	-63.04	-88.31	-1.106
Isoliquiritin apioside	-55.95	-79.92	-1.435
Oroxin B	-50.99	-58.91	-1.214
JI-101	-19.42	-32.52	-0.647
Poliumoside	-41.57	-71.24	-0.77
Clovamide	-20.04	-31.2	-0.771
Rhodiosin	-62.54	-77.98	-1.455
4,5-Dicaffeoylquinic acid	-36.9	-47.7	-0.997
Astringin	-19.67	-29.16	-0.678
Hispolon	-29.61	-38.65	-1.851
Narirutin	-48.29	-69.07	-1.178
1,3-Dicaffeoylquinic acid	-9.56	-34.56	-0.258

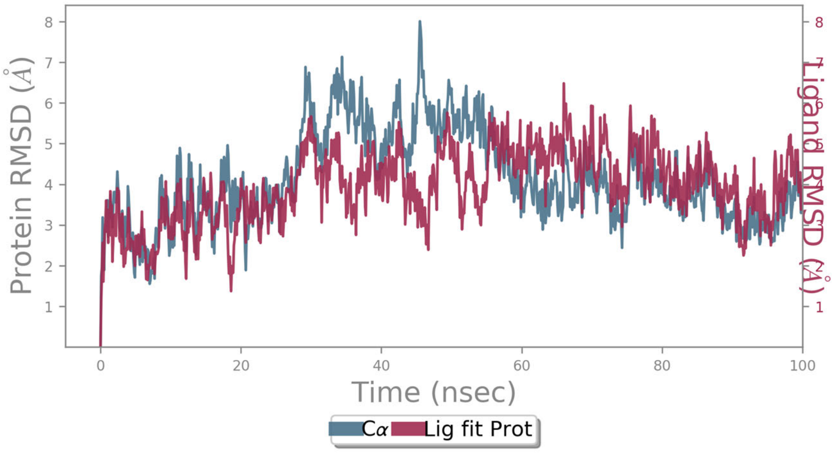

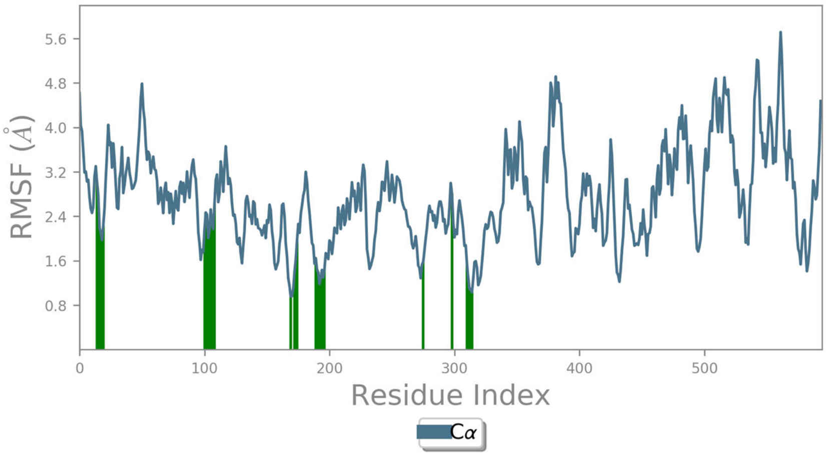

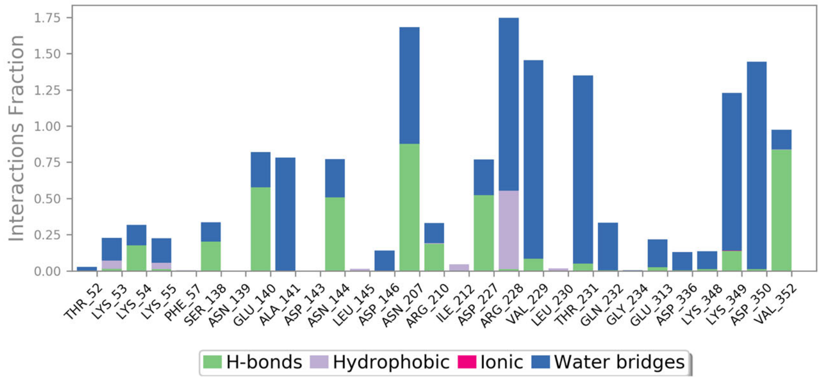

https://doi.org/10.3390/ph19050752

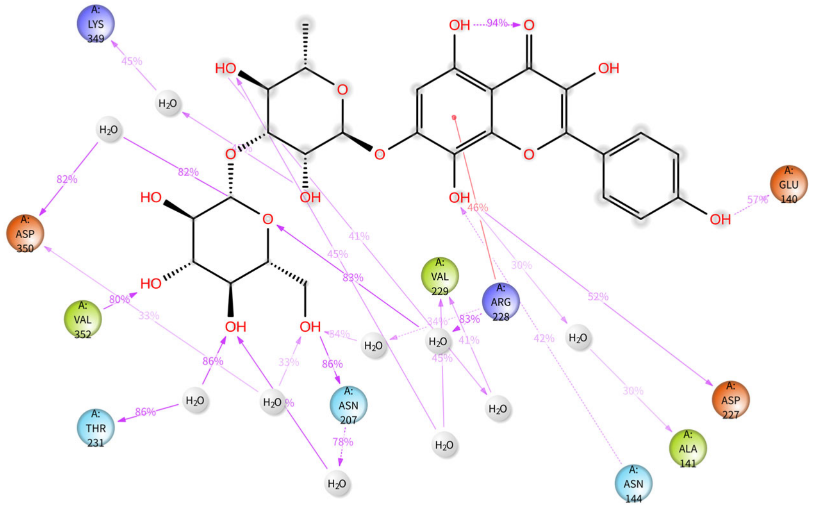

Figure S2. RMSD, RMSF, protein–ligand contacts and ligand–protein contacts.

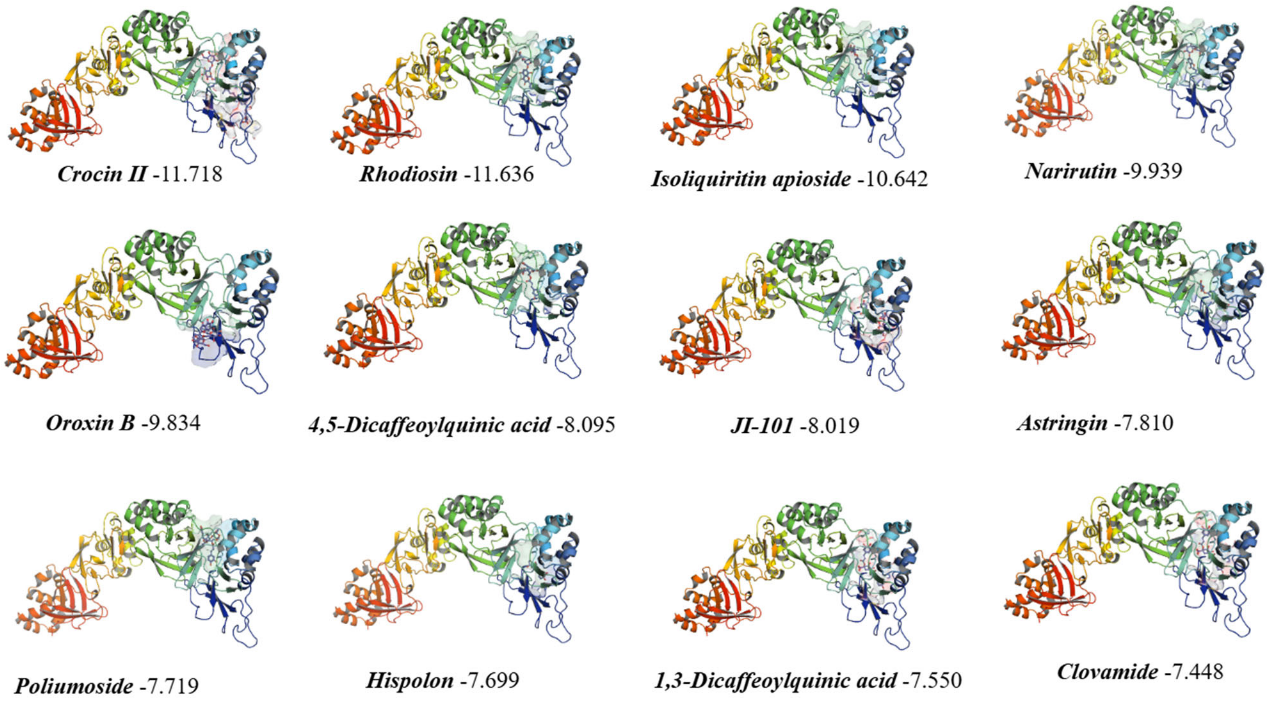

Figure S3. Schematic diagram of receptor–ligand interactions for all key candidate molecules.

https://doi.org/10.3390/ph19050752

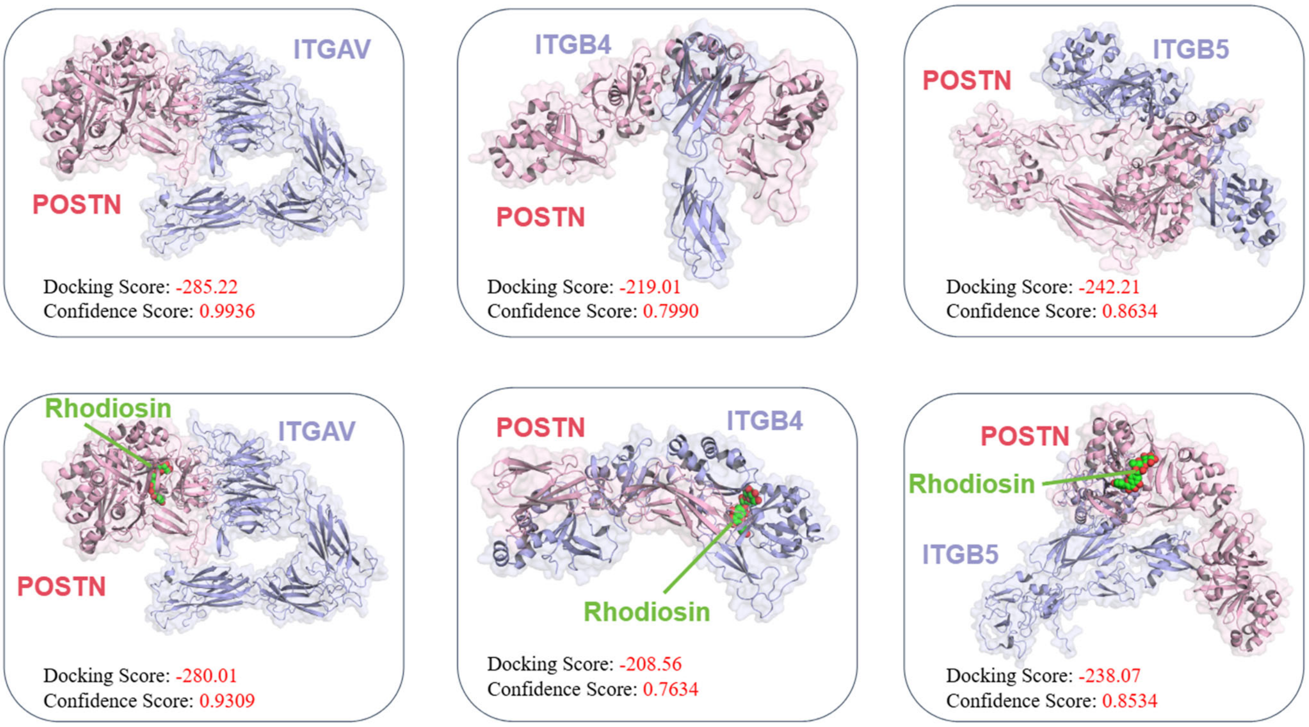

Figure S4. Rhodiosin may exert its effects by blocking the binding of POSTN to integrins.

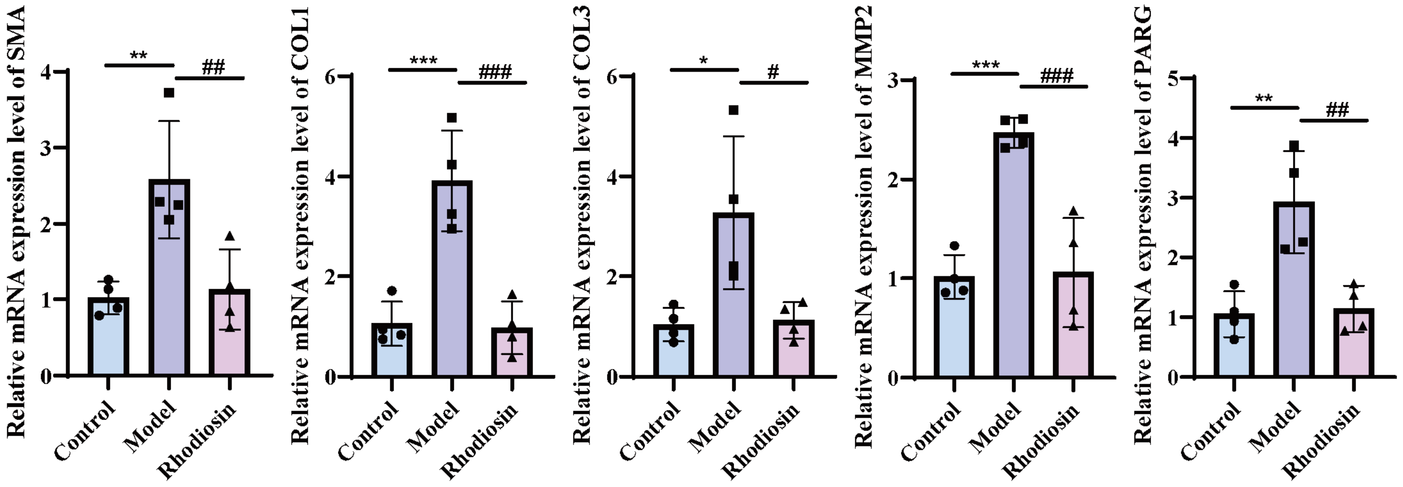

Figure S5. Rhodiosin could contribute to the inhibition of HSC activation and fibrosis formation. **P* < 0.05, ***P* < 0.01, and ****P* < 0.001 *vs*. model, #*P* < 0.05, ##*P* < 0.01, and ###*P* < 0.001 *vs*. Rhodiosin, n=4, analyzed via one-way analysis of variance (ANOVA).
